# Interleukin-10: A Key Cytokine in Depression?

**DOI:** 10.1155/2009/187894

**Published:** 2009-08-16

**Authors:** Susana Roque, Margarida Correia-Neves, Ana Raquel Mesquita, Joana Almeida Palha, Nuno Sousa

**Affiliations:** Life and Health Sciences Research Institute, School of Health Sciences, University of Minho, Campus Gualtar, 4710-057 Braga, Portugal

## Abstract

An increasing body of evidence implicates proinflammatory cytokines in psychiatric disorders, namely, in depression. Of notice, recent studies showed that anti-inflammatory cytokines, such as IL-10, also modulate depressive-like behavior. In this article, we propose that the anti-inflammatory cytokine IL-10 is a putative link between two of the most widely reported phenomenon observed in depressed patients: the disruption of the hypothalamic-pituitary-adrenal axis and the imbalanced production of cytokines. If so, IL-10 might represent a novel target for antidepressant therapy.

## 1. Introduction

The establishment of a bidirectional interaction between the immune and central nervous systems is one of the most remarkable findings of the last decades. These two systems accomplish extremely different functions, but a growing body of evidence shows that both share common mediators, including cytokines and their receptors. Therefore, the altered expression of these molecules, triggered by one of the systems, might influence the other in a reciprocal way [1]. As a consequence, a disruption in this cross-talk has been causally implicated in neuropathological features associated with several psychiatric disorders, in particular depression [2].

Major depression is a global public-health problem and a leading cause of disability worldwide, representing the fourth contributor to the global burden of disease in 2000 (in terms of DALYs-Disability Adjusted Life Years) [3]. Depression is a heterogeneous disorder with a variable set of symptoms, diverse disease courses, and inconsistent responses to treatment. The etiology of depression is still controversial, and several theories have emerged to explain it. Taking into account the communication between the immune and central nervous systems, Smith, in the early 1990s, proposed a role for cytokines in depression [4]. The “cytokine theory of depression”, that has been widely studied in the last two decades, proposes that enhanced production of proinflammatory cytokines is associated with the pathogenesis of depression. Indeed, several studies show a significant increase in the production of proinflammatory cytokines (namely, IL-6, IL-1*β*, IFN-*γ*, and TNF) among depressed patients [5–8]. Moreover, immunotherapy based on the administration of IFN-*α* and IL-2, which is frequently used as part of the treatment against chronic hepatitis C and certain cancers, has been associated with depressed mood and symptoms of cognitive impairment and fatigue [8–10]. The fact that the symptoms associated with depression disappear almost immediately after termination of cytokine administration supports a causal role for cytokines in the disease; moreover, these symptoms can also be relieved by the administration of antidepressant drugs [11].

The “cytokine theory of depression” has also been supported by a large set of results in animals models. Research using these models demonstrated that alteration in the proinflammatory cytokine milieu can lead to behavioral changes overlapping with those found in depressed patients, including anhedonia, decreased activity, cognitive dysfunction, and altered sleep patterns [12]. In further support of this link, mice exposed to a chronic mild stress (CMS) protocol, which induces symptoms of depressive-like behavior, show increased levels of IL-1 in the hippocampus [13]. Furthermore, the depressive-like behavior observed after CMS can be mimicked by chronic administration of IL-1 [13] and, when mice lacking the expression of IL-1 receptor are exposed to the same CMS paradigm, the behavioral changes do not occur [13]. Interestingly, mice unable to express the receptor for TNF, one of the cytokines more consistently upregulated in depression, show decreased immobility in the forced swim test (FST), that is, decreased signs of depressive-like behavior in a test that assesses learned helplessness and is considered one of the gold standard tests to evaluate depression in rodents [14]. These observations suggest that TNF activation also mediates depressive-like behavior in mice.

Although most studies on the “cytokine theory of depression” are centered on increased levels of proinflammatory cytokines, the role of anti-inflammatory cytokines has been recently analyzed. Of notice, IL-10, one of the most important anti-inflammatory cytokines, proved to be relevant in depression [15]. Specifically, mice lacking the expression of IL-10 (IL-10KO) show a decreased latency to immobility and longer immobilization time than wild-type (WT) mice in the FST [15]. These results demonstrate that IL-10KO mice display increased helplessness, a recognized sign of depressive-like behavior in rodents. Remarkably, administration of IL-10 is able to revert the depressive-like phenotype observed in the IL-10KO animals [15]. In further support of a role for IL-10 in depression, transgenic mice overexpressing this cytokine show a decreased depressive-like behavior in the FST in comparison with WT animals [15]. In addition to the increased helplessness in mice that lack the expression of IL-10, other studies also reported that modulation of IL-10 impacts on psychophysiological alterations frequently observed in depression. Among these is the impairment in sleep behavior [16]. Interestingly, IL-10KO mice show alterations in the sleep pattern, and the exogenous administration of IL-10 modulates sleep behavior [17–19]. 

Another feature commonly associated with depression is altered pain perception [20]. Tu and coworkers described, using the IL-10KO mouse model, that the absence of this anti-inflammatory cytokine is associated with decreased nociception (i.e., the ability to sense painful stimuli). This study showed that IL-10KO mice have an increased latency time to paw licking (the time that mice spend to avoid a heat stimulus by moving away and lick the paw) compared with WT mice, and this result was confirmed by the blockage of IL-10 in WT mice [21]. This constitutes an additional evidence that IL-10 can also be involved in the common biological pathways shared by pain and depression. 

The studies described above show that modulation of IL-10 impacts on several symptoms associated with depression, namely, helplessness, sleep disturbances, and pain perception. Noticeable, administration of IL-10 can modulate these symptoms which suggests a putative antidepressant effect of the anti-inflammatory cytokine IL-10. Of relevance for this hypothesis is the observation, in depressed patients and also in animal models of depression, of increased IL-10 levels after treatment with several classes of antidepressants [22–25]. 

Although it is becoming clear that modulation of IL-10 can lead to changes in the normal behavior of humans and animals, the mechanisms behind these alterations remain to be elucidated. One might envisage several pathways through which IL-10 influences behavior. One of the most obvious hypotheses is the impact this anti-inflammatory cytokine may have on the levels of proinflammatory cytokines. In fact, it is admissible that the absence of IL-10, in IL-10KO mice, leads to an increased production of proinflammatory cytokines that could, in accordance with the “cytokine theory of depression”, trigger depression. This mechanism could also explain the abrogation of the depressive-like behavior in IL-10KO mice upon IL-10 administration [15], as IL-10 is known to inhibit the expression of proinflammatory cytokines [26]. However, this possibility is not supported by our results, as we were unable to detect two of the most relevant proinflammatory cytokines (IFN-*γ* and TNF) in the serum of IL-10KO mice [15]. More studies are certainly necessary to clearly define how the lack of IL-10 impacts on the production of proinflammatory cytokines as it is possible that subtle (below currently detection limits) changes in the concentration of these cytokines are present in the serum of IL-10KO mice, and/or that the absence of IL-10 induces the upregulation of proinflammatory cytokines in specific brain regions, which may not be reflected in the serum.

The observations that systemic IL-10 administration to WT animals induces alterations in their normal behavior (e.g., increased motor activity and abnormal exploratory patterns) [27] and that the same protocol reverts the depressive-like behavior observed in IL-10KO mice [15] indicate that peripheral IL-10 has an effect in the central nervous system. However, it is not clear how this cytokine acts within the central nervous system particularly since peripheral IL-10 does not seem to cross the intact blood-brain barrier, at least in detectable amounts [28]. It is possible that IL-10, like other cytokines, acts directly in the brain through regions devoided of blood-brain barrier, such as the circumventricular organs and the choroid plexus, or by activating vagal afferent fibers that can transmit cytokine signals to specific brain nuclei [5]. Moreover, IL-10 has been detected in normal human, rat, and mouse brains [29]. Possible sites of expression are the endothelial cells of the brain capillaries that irrigate the brain, the choroid plexus (from where it would be secreted into the cerebrospinal fluid), and also microglia and astrocytes [29–31]. Of interest, a recent study showed that several immune mediators are produced by the choroid plexus in response to systemic inflammatory stimuli [32]. In addition, the IL-10 receptor has been indentified in microglia, astrocytes [29, 30], and oligodendrocytes [29, 30, 33]; probably because of a general distribution, Ward and coworkers found expression of the receptor in all five brain regions they analyzed (cortex, cerebellum, hippocampus, hypothalamus, and pituitary) [34]. 

A direct action of IL-10 within the central nervous system might be mediated by its role in regulating cell survival. IL-10 has been shown to prevent cell death of glial cells [33, 35, 36] and to increase survival of cerebellar neurons [37]. Since an increase in neuronal apoptosis in the hippocampus has been associated with depression in animal models [38], this putative role of IL-10 in increasing neuronal survival should be investigated as a potential mechanisms of action in preventing depressive-like behavior. 

The observation that IL-10KO mice show an increase in the adrenal and a decrease in the thymus relative weights [15] offers another alternative mechanism for the action of IL-10 in depression: the modulation of the hypothalamic-pituitary-adrenal (HPA) axis. Indeed, it was shown that, even in basal conditions, IL-10KO mice have higher levels of corticosterone and that in the presence of a stressor the increase of this hormone is even more accentuated than in WT mice [39]. These findings strongly suggest that IL-10 may have a regulatory effect in the HPA axis. One of the most consistent neurobiological alterations in depressed subjects is the hyperactivity of the HPA axis which is associated with impaired HPA axis glucocorticoid feedback sensitivity (glucocorticoid resistance) [40]. Thus, it is plausible that IL-10 modulation of depressive-like behavior is exerted through regulation of the HPA axis. In accordance, IL-10 is able to suppress, in a dose dependent manner, adrenocorticotropic hormone-(ACTH-) induced steroid production in adrenal cells [41]. This effect appears to be exerted through downregulation of enzymes responsible for the biosynthetic pathway of corticosterone [21, 41]. Interestingly, IL-10 receptor was shown to be expressed in the zona fasciculata (the region responsible for the production of glucocorticoids) of the mouse adrenal gland [41]. Accordingly, in vivo studies demonstrated that, under basal conditions, IL-10KO mice have clear signs of HPA axis activation, such as increased adrenal glands, decreased thymus relative weight, and higher levels of corticosterone [15, 39]. These findings strongly suggest that IL-10 exerts a negative regulation on corticosterone production by the adrenal gland [41]. A microarray analysis of adrenal, pituitary, and hypothalamic neural cells treated with IL-10 clearly showed that this anti-inflammatory cytokine plays a pivotal role in the regulation of several genes of the HPA axis [42]. Of notice, murine pituitary cells were the first identified nonimmune-related sources of IL-10 [43], and the presence of IL-10 was also found in human pituitary [44]. Paradoxically, in the hypothalamus and pituitary, IL-10 seems to have the same effect already described for some proinflammatory cytokines [45], which is a positive regulation of corticotrophin releasing factor (CRF) and ACTH production, respectively [43, 46]. It should be noticed that while the studies in the hypothalamus and pituitary were only performed in vitro, the inhibitory action of IL-10 in corticosterone production by the adrenal gland is a more consistent result as it is based on in vitro and in vivo studies. Taking into account that depression is often associated with hyperactivity of the HPA axis [40], we hypothesize that IL-10 has a pivotal role in the modulation of the HPA axis homeostasis, which is likely to have an impact on the etiology of depression. 

**Figure 1:**
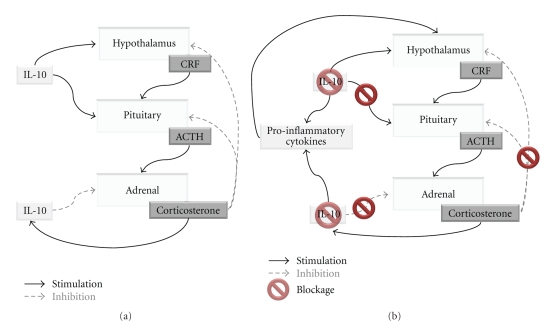
Schematic representation of the link between IL-10 and HPA axis. (a) in a basal situation and (b) during depression.

Increases in glucocorticoid levels can occur daily in response to several factors. This glucocorticoid increase leads to an enhanced production of IL-10 [47] which, in normal situations, together with glucocorticoids, inhibits the activity of the HPA axis (Figure 1(a)). However, if these raises in glucocorticoid levels become too frequent, cells might develop “resistance to glucocorticoid action” [40, 48]. In fact, impaired negative feedback regulation of HPA axis function is a hallmark of major depression and is reflected by decreased responsiveness to glucocorticoids [40]. Of notice, resistance to glucocorticoids was also described in IL-10 producing cells [48]. The glucocorticoid resistance leads to a decrease in the production of IL-10 which might impact on the negative regulation of corticosterone production by the adrenal glands. In addition, decreased IL-10 can promote an imbalance in the cytokine milieu that would further activate the HPA axis (Figure 1(b)). This hypothesis fits well with the observations that, in certain circumstances, glucocorticoids trigger a proinflammatory action [49].

In the hypothesis outlined here we proposed IL-10 as an important link between the changes in hormonal and cytokine milieus that are of recognized relevance for depression. In the future, studies in molecules that might represent a link between the modulation of neural, endocrine, and immune systems are extremely important to further understand the etiology of depression. The cross-talk between these systems, mediated by IL-10, may become a target for novel antidepressant therapies.
